# The Conselice Study of Brain Ageing

**DOI:** 10.1186/1742-4933-7-S1-S2

**Published:** 2010-12-16

**Authors:** Giovanni Ravaglia, Paola Forti

**Affiliations:** 1 Department of Internal Medicine, Ageing, and Nephrology, University of Bologna, University Hospital S. Orsola-Malpighi, Via Massarenti 9, 40138 Bologna, Italy

## Abstract

Among the age-related diseases, the development of cognitive impairments, in particular dementia, is the most devastating for the individual and has great social and healthcare costs. Accurate information is needed about the prevalence and incidence of cognitive disorders and the physiology of the ageing brain. In particular, only scant data are available about the relationship between ageing, cognitive status and nutritional factors. In order to address these issues we planned the Conselice Study of Brain Ageing, a longitudinal study of physiologic and pathologic brain ageing. The center involved in the study was the municipality of Conselice, Ravenna province, in the Northern Italian region Emilia-Romagna. A total of 1016 subjects aged 65 and over was enrolled at baseline. Information about cognitive status at 4-years of follow-up was collected for 940 of them. These data have been used to estimate prevalence and incidence of dementia in the elderly Italian population and to investigate the possible role of baseline blood homocysteine as risk factors for dementia.

## Introduction

Among the age-related diseases, dementia is the most devastating for the individual and the one with the greatest social and healthcare costs. Therefore, a better understanding of potentially modifiable risk factors for cognitive impairment of older age and early identification of individuals at increased risk of dementia is a high priority. The Conselice Study of Brain Ageing was specifically planned to address the following specific objects:

1) collection of epidemiologic information about the prevalence and incidence of dementia in a sample representative of the elderly Northern Italian population;

2) investigation of dementia risk factors, with particular focus on potentially reversible conditions such as hyperhomocysteinemia;

3) creation of a large biomedical bank of data (including lifestyle, nutritional, physical and cognitive status, and laboratory values) and biological material available for further multidisciplinary studies of ageing.

## Design and methods of the conselice study of brain ageing

The study was conducted by the Centre for the Study of Fisiopathology of Ageing, University of Bologna, in cooperation with the Conselice Municipality, the Ravenna local health unit (Azienda Unità Sanitaria Locale (A.U.S.L) of Ravenna, Italy) and Conselice general practitioners.

### Study population and sample size

The centre involved in the study is the municipality of Conselice, in the Ravenna Province, Emilia Romagna region. This municipality is representative of the Emilia Romagna region in terms of geographic distribution, sociodemographic, and life-style factors. It has a stable population with a very low migration rate (less than 0.2% of people over 64 years had moved elsewhere in the five years preceding the study). Conselice area was a rural one, and only during the last two decades has been transformed into an urban area, with middle to high socio-economic status. However, elderly inhabitants of this area were raised and lived in a rural environment most of their life.

All the individuals aged 65 years and older who at January 1, 1999, according to the municipal registry office, were residing in Conselice were considered eligible for the study. Between 1999-2000 1016 of 1353 eligible individuals (75%) were evaluated: 453 men and 563 women, mean age 74.6 ± 7.1 years and with on average 4.6 ± 2.4 years of education. Blood samples were obtained from 985 participants. Refusers did not significantly differ from participants for gender, age and education. Between 2003-2004, 751 of the 864 survivors of the baseline cohort were re-assessed. Moreover, information adequate for a cognitive status diagnosis was collected for 65 of the 113 refusers and 124 of the 152 individuals who deceased before follow-up.

All participants gave their written, informed consent. The study protocol was approved by the local ethical board.

### Evaluation procedures

The same procedures and diagnostic tests were used at baseline and at the follow-up examination. At each examination, evaluation included two phases:

1) extensive screening of all participants in order to characterize their actual biomedical, cognitive and socio-functional status;

2) neuropsychological testing and neuroradiological assessment for all suspect cases of cognitive impairment identified at the screening phase in order to formulate a final diagnosis of dementia.

The screening included a personal interview, a clinical evaluation and fasting venous blood drawing:

a) Personal interview. A comprehensive standardized questionnaire was administered to each subject including information on socio-demographic characteristics, lifestyle habits, medical history and current use of medications. Whenever available, medical records were reviewed. If the subject was unable to answer because of physical or mental impairments, information was obtained from a proxy informant (relatives and the subject’s general pratictioner). Diagnoses were recorded according to the International Classification of Diseases, 9th revision (ICD-9). The leisure time physical activity assessment within the past year was done using the Paffenbarger Physical Activity Questionnaire interviewer-administered [1].

Subjects residing in institutions or unable to leave their home were visited at their dwelling. For subjects who did not participate to the follow-up examination, telephone information about their cognitive and functional status was sought. All deaths in the baseline cohort during follow-up time were identied from the municipality registry office. Information regarding cognitive status in the last six months prior to death was collected from all available sources (relatives, medical charts, general pratictioners).

b) Clinical Evaluation. All participants underwent a standardized and extensive medical examination also including standardized anthropometric measurements, the Tinetti test for mobility and balance, Basic and Instrumental Activities of Daily Living, neuropsychological screening using the Italian version of Folestein’s Mini Mental State Examination (MMSE), the Clock Drawing Test, and the Yesavage’s Geriatric Depression Scale (see [2] for full details and references about the instruments used for the assessment).

c) Venous blood drawing after an overnight fast. The blood samples collected at baseline were partly used for routine blood analysis (including full blood count, ESR, glucose, lipids, renal, liver, and thyroid function, fibrinogen, CRP, folate, vitamin B12) and partly stored as serum, plasma and DNA aliquots at -70°. Total homocysteine was measured on frozen plasma sample by fully automatized fluorescence polarization immunoassay (see [3] for full details). Further neuropsychological evaluation of the subjects with suspect cognitive impairment at the screening phase was performed using the the Mental Deterioration Battery [4] and scheduling a non-contrast computed tomography brain scan for all subjects with MMSE < 24/30. For persons scoring below ten on the MMSE further neuropsychological testing was not deemed necessary. The physicians responsible for the neuropsychological examination discussed all possible cognitive impairment cases with a senior physician and final diagnoses were made after reviewing all available medical, laboratory, and neuroradiological data. If additional information were needed, the patient’s general pratictioner was contacted. Dementia was defined by DSM IV criteria [5], Alzheimer’s disease (AD) by NINCDS-ADRDA criteria [6] and Vascular Dementia (VaD) by NINDS-AIREN criteria [7]. The diagnosis of dementia was made only in subjects with duration of cognitive decline of at least six months. Subjects affected by major sensory or motory deficits, by severe depression (Geriatric Depression Scale score > 20), or by other psychiatric disease hampering a reliable cognitive assessment were deemed “cognitively unclassifiable”.

## Prevalence and incidence of dementia in the CSBA

Most of the current knowledge about of dementia epidemiology in Italy is based on prevalence data, which show variations due to differences in case ascertainment procedures, diagnostic criteria, and age distribution of the population studied. Population-based longitudinal studies provide the best estimate of the problem dimension as well as identification of risk and protective factors, but are expensive and time-consuming. Before the conclusion of the CSBA first follow-up, the only available data on dementia incidence in Italy were those provided almost ten years ago by the Italian Longitudinal Study of Aging (ILSA) [8], reporting an incidence rate of 12.5 per 1,000 person-years for dementia, 6.6 per 1,000 person-years for Alzheimer Disease, and 3.3 per 1,000 person-years for vascular dementia (data standardized to the 1995 Italian population).

## Epidemiology of dementia in the CSBA cohort

Overall prevalence of dementia in the CSBA cohort, calculated on the 1016 subjects who completed the protocol at baseline, was 5.9% (95%CI: 4.2 – 8.0) [9]. These figures are similar to those from ILSA [8] and other European population-based studies [10]. Our study confirmed AD as the most frequent type of dementia (50% of all cases), whereas our prevalence rate for VaD (45%) was almost doubled with respect to previous estimates [11]. A possible methodologic explanation is that, differently from previous studies, our diagnoses of dementia subtypes incorporate information from neuroradiologic data, and NINDS-AIREN classification of VaD includes cases of dementia resulting from brain infarcts as well as from small-vessel disease. Another important finding from the prevalence phase of CSBA is the association found between education and dementia prevalence [9]. According to the “brain reserve hypothesis”, education may provide a reserve of brain capacity able to compensate for brain structural damages [12]. It may be tempting to hypothesize a reduction in dementia prevalence as a result of the progress of education among western populations. A number of methodological problems, however, particularly culture-fair dementia diagnostic procedures, need to be solved before research in this area can generate reliable estimates.

According to data from the follow-up examination at four-year of the CSBA cohort [13], incidence rates per 1,000 person-years were 37.8 (95% CI = 30.0 – 47.7) for all-cause dementia, 23.8 (95% CI = 17.3 – 31.7) for AD, and 11.0 (95% CI = 7.2 – 16.9) for VaD. These estimates are substantially higher than those previously reported in the ILSA cohort. Table 1 details incidence of dementia and its subtypes by age group in the CSBA cohort and also reports estimates for the expected number of new cases in the Italian population as calculated from national demographic projections for 2020 (ISTAT 2004). On the basis of CSBA data, in 2020 our country might have to face about 584,000 new dementia cases versus the 230,000 new cases predicted applying the ILSA rates.

**Table 1:** Incidence rates of dementia in the Conselice Study of Brain Aging and corresponding projections in 2020 for the Italian population

Age Groups, Years	Findings in 2003-2004 Rate per 1,000 person-years	Projection in 2020: No. of expected new cases in Italy
All dementias		
65 – 69	8.6 [3.9 – 19.2]	
70 – 74	23.3 [15.2 – 35.9]	
75 – 79	45.02 [32.2 – 63.0]	
80 – 84	55.1 [36.7 – 83.0]	
85 – 94	111.8 [78.7 – 159.0]	
All ages	37.8 [30.0 – 47.7]*	584,575
Alzheimer’s Disease		
65 – 74	11.3 [7.1 – 17.9]	
75 – 84	28.1 [20.0 – 39.6]	
85 – 94	75.8 [49.4 – 116.2]	
All ages	23.8 [17.3 – 31.7]*	378,310
Vascular Dementia		
65 – 74	5.0 [2.5 – 10.0]	
75 – 84	16.2 [10.3 – 25.4]	
85 – 94	25.2 [12.0 – 53.0]	
All ages	11.0 [7.2 – 16.9]*	165,388

* standardized to the 2003 Italian population

95% confidence interval in brackets

## Homocysteine as a risk factor for cognitive impairment and dementia

Homocysteine is a sulfur-aminoacid whose blood levels, in elderly people are frequently increased because of age, physiological decrease in renal function, and deficits of B vitamins, mainly folate and vitamin B12 [14]. Homocysteine is currently recognized as a predictor of vascular disease [15], but evidence is growing for its possible role as a risk factor for dementia in the elderly [16]. Mechanisms suggested for a causal role of hyperhomocysteinemia in the etiopathogenesis of brain damage are: vascular damage, impaired brain methylation reactions related to folate and vitamin B12 deficits, and direct neurotoxic effects. Using data from the prevalence phase of the CSBA, we reported for the first time that, in healthy non-demented elderly community-dwellers, elevated plasma homocysteine has an independent, graded association with concurrent cognitive impairment as measured with the MMSE [3]. Moreover, in the CSBA cohort, hyperhomocysteinemia (defined as plasma tHcy > 15 μmol/L), was associated with an increasing risk of developing dementia (HR 2.08, 95%CI 1.31-3.30) and AD (HR 2.11, 95%CI 1.19-3.76) at follow-up [17]. Independently of hyperhomocysteinemia and other confounders, low folate levels were also associated with an increased risk of both dementia and AD whereas no association was found for vitamin B12. As shown in Table 2, which reports risk of any dementia and AD across quartiles of plasma tHCy and serum folate, the associations exhibited a dose-effect trend. Our findings cannot be used as a basis for treatment recommendations. However, they prompt realization of clinical trials in humans aimed to verify whether interventions that restore folate status and reduce plasma homocysteine can lower the risk of dementia and AD in the Italian population.

**Table 2:** Risk of any dementia and Alzheimer Disease by quartiles of plasma total homocysteine and serum folate

	Any Dementia		AD	
	HR [95% Confidence Interval]	P value	HR (95% Confidence Interval)	P value

Quartiles of plasma total homocysteine, μmol/L				
< 10.1	1.00		1.00	
10.1 to 12.5	1.73 [0.87 – 3.46]	0.117	2.43 [0.96 – 6.13]	0.062
12.6 to 15.0	2.02 [0.96 – 4.23]	0.060	2.43 [0.96 – 6.13]	0.060
> 15.00	3.55 [1.72 – 7.29]	0.001	4.33 [1.73 – 10.8]	0.002
Quartiles of serum folate, nmol/L				
< 8.9	2.22 [1.21 – 4.05]	0.010	2.04 [1.02 – 4.09]	0.045
8.9 to 11.8	1.83 [1.00 – 3.34]	0.050	1.30 [0.62 – 2.72]	0.484
11.9 to 15.2	1.16 [0.60 – 2.24]	0.064	0.66 [0.29 – 1.54]	0.340
> 15.2	1.00		1.00	

Data are for 816 subjects; the number of incident dementia cases was 112 for any dementia and 70 for Alzheimer’s Disease (AD).

Hazard Ratios (HR) are adjusted for age, gender, education, APOE genotype, creatinine, serum levels of folate and vitamin B12, stroke, smoking status, diabetes, hypertension, cardiovascular disease, and body-mass-index.

## Other modifiable dementia risk factors identified in the Conselice Study of Brain Ageing

The CSBA database is proving an invaluable resource for investigations of other modifiable dementia risk factors such as the antioxidant vitamin E. A study of the effect of vitamin E status on dementia risk in this cohort [18] did not confirm previous reports of a beneficial association with plasma alpha-tocopherol, which is the most abundant vitamin E form in the human body, the one with the highest biological activity, and also the most extensively investigated. However, a U-shaped association, with lower risk for intermediate tertiles, was found for incident dementia with γ-tocopherol (Hazard ratio: 0.42; 95%CI: 0.22-0.84). The only investigation including measuring of plasma γ-tocopherol has a cross-sectional design and reported no association of this tocopherol form with concurrent cognitive performance [19]. By contrast, the inverse association found in the CSBA cohort between risk of incident dementia and plasma γ-tocopherol agrees with results from a study suggesting that dietary γ-tocopherol may have a protective role against dementia [20]. Although a less powerful chain-breaking antioxidant than α-tocopherol, γ-tocopherol is superior as a scavenger of reactive nitrogen species, which are thought to significant contribute to neurodegenerative diseases via oxidative damage [21]. Additionally, γ-tocopherol has anti-inflammatory properties [22] that may be important in dementia prevention. It is not clear why, in our study, the beneficial effect of γ-tocopherol was evident only for the middle tertile. A possible explanation is that higher γ-tocopherol status may be associated with unhealthy dietary behaviours, such as higher intakes of saturated and trans-unsaturated fats [21].

Another relevant finding is the association between VaD risk and physical activity [23]. Longitudinal investigations of the effect of physical activity on dementia risk in elderly persons are few in number and produced inconsistent results. A lower risk of all-cause dementia and Alzheimer’s dementia (AD) among subjects regularly practising low-to-medium intensity physical activities was found in some population-based studies [24-28] but not in others [29-32]. Moreover, only a few investigations examined the effect of physical activity on vascular dementia (VaD) risk [24,25,27,32] and none reported an association.

Longitudinal investigations of the effect of physical activity on dementia risk in elderly persons are fewer in number and produced inconsistent results, especially as far as VaD is concerned. In the CSBA cohort, VaD risk was significantly lower for the upper tertiles of walking (Hazard Ratio [HR] 0.27, 95%CI 0.12 to 0.63), moderate (HR 0.29, 95%CI 0.12 to 0.66), and total physical activity (HR 0.24, 95% 0.11 to 0.56) compared to the corresponding lowest tertile. The association persisted after accounting for vascular risk factors and overall health status. The following, not mutually exclusive, hypotheses have been proposed to explain how cognition may benefit from physical activity [33,34]. First, physical activity may improve cerebral blood flow and lower the risk of cerebrovascular disease and. Second, physical activity may stimulate synaptic plasticity, secretion of trophic factors, neurotransmitter synthesis, and neurogenesis, providing cognitive reserves against brain damage. Third, physical activity may decrease secrection of brain-toxic stress hormones like cortisol. Finally, more than from exercise itself, the beneficial effects of physical activity on cognition might result, all or in part, from the mental and social stimulation related to an active lifestyle. It is important to note that, in terms of lowering VaD risk, an easy-to-perform moderate activity like walking provided the same benefits of other, more demanding activities of similar intensity, and being in the upper tertiles of total weekly energy expenditure did not offered any specific additional advantage. This finding may be important for future studies attempting to define strategies for prevention of cognitive impairment in the elderly.

## List of abbreviations

AD: Alzheimer’s Disease; CRP: C-reactive protein; CSBA: Conselice Study of Brain Ageing; DSM IV: Diagnostic and Statistical Manual of Mental Disorders IV edition; ESR: erythrocyte sedimentation rate; HR [95%CI]: Hazard ratio and corresponding 95% Confidence Interval; ILSA: Italian Longitudinal Study on Aging; MMSE: Mini Mental State Examination; NINCDS-ADRDA, National Institute of Neurological and Communicative Disorders and Stroke and Alzheimer's Disease and Related Disorders Association; NINDS-AIREN: National Institute of Neurological Disorders and Stroke and Association Internationale pour la Recherche et l'Enseignement en Neurosciences; tHcy: total homocysteine; VaD: Vascular Dementia;

## Competing interests

The authors declare that they have no competing interests.

## Authors' contributions

GR and PF conceived of the Conselice Study of Brain Ageing, participated in its design and coordination and drafted the present manuscript. Both authors read and approved the final manuscript.
